# In response to the Letter to the Editor by Ould Setti and Ould Setti RE: ‘Non-specific Effects of vaccines and stunting: Timing May Be Essential’

**DOI:** 10.1016/j.ebiom.2021.103606

**Published:** 2021-10-19

**Authors:** Mike L.T. Berendsen, Jeroen Smits, Mihai G. Netea, Andre van der Ven

**Affiliations:** a Department of Internal Medicine and Radboud Center for Infectious Diseases, Radboud University Medical Center, Nijmegen, the Netherlands; b Global Data Lab, Institute for Management Research, Radboud University, Nijmegen, the Netherlands; c Department for Genomics & Immunoregulation, Life and Medical Sciences Institute (LIMES), University of Bonn, Bonn, Germany

We welcome the interest of Ould Setti and Ould Setti regarding our study that aimed to evaluate the effect of timing of infant vaccination with Bacillus Calmette-Guérin (BCG), diphtheria-tetanus-pertussis (DTP)1, and measles vaccine (MV) on stunting and hemoglobin concentration using survey data on 368.450 children from 33 Sub-Saharan African countries [1]. The authors raise the question of a possible bias influencing our conclusions in relation to two factors: 1) parental adverse childhood experiences, notably neglect; 2) neonatal and serious infancy infections.

The observational nature of our study comes with its limitations. As in any observational study, careful assessment and adjustment of possible confounding should take place to prevent biased outcomes. As described by Ould Setti and Ould Setti, both adverse parenting as-well-as neonatal and serious infancy infections could be associated with the delay of vaccination and the odds of stunting. They, therefore, fulfill the classic requirements of a confounder and should be controlled for. Although we performed an extensive adjustment for a range of covariates at the child's individual level, the household level, and the regional level, data directly measuring these factors was not available in the database.

Nevertheless, there are several indications that render possible bias due to these factors unlikely. For adverse parenting, we included several proxies as covariates into the analyses. Although these proxies cannot fully control for a diverse factor such as adverse parenting, they do reveal patterns that are incompatible with adverse parenting being the cause of our found associations. Children who were vaccinated, both early and late in infancy, scored better on the included proxies than their unvaccinated counterparts, while their odds of stunting were opposite. As displayed in Supplementary Table 3 of the manuscript, children who had received a BCG vaccination >7 months after birth had more often received other vaccinations or vitamin A supplementation than BCG unvaccinated children. They were also more often delivered in a hospital and their mothers had received more years of education. All these findings strongly argue against poor parenting in the case of these children. If parental neglect would have caused the higher odds of stunting in children vaccinated later in infancy, we would have expected to find poorer outcomes among this group for these proxies as well. In addition, children suffering most of parental neglect would have been unvaccinated, rather than being vaccinated late in infancy.

Delayed vaccinations as a result of contraindications due to neonatal and serious infancy infections would result in the healthiest children receiving their vaccines early, leaving a frail group that suffered multiple infections to have delayed or no vaccination. Comparing those already vaccinated with those who are (still) unvaccinated, creates a healthy vaccinee bias. This is a difficult factor to control for since it would require longitudinal data on infections or nutritional status for all children at each recommended age of vaccination. This information was not available but, similar to parental neglect, it would be expected that children who would have suffered the most infections, would not receive vaccination at all. This does not correspond to the lower odds seen for unvaccinated children compared to children vaccinated later in infancy. In addition, in a more recent study using similar survey data we found no time-dependent association between BCG and malaria infection, but did find a time-dependent association of BCG with stunting [2]. The contradiction between these two outcomes would advocate against a healthy vaccinee bias as a cause for the time-dependency.

Ould Setti and Ould Setti also propose an adjustment by including timing of other vaccinations, most likely as a delayed BCG vaccine with correctly timed DTP1 and/or other vaccines could be indicative of delay due to contraindications. We did perform a combined timing analysis with BCG and DTP1 to study the robustness of the associations (Supplementary Figure 3 of the manuscript). Both vaccines still had a time-dependent association with stunting.

Although we cannot completely rule out residual confounding, as discussed in the manuscript, we believe the issues raised by Ould Setti and Ould Setti are unlikely to have caused the found associations between timing of BCG, DTP1 and MV vaccination and stunting.

## Contributors

All authors conceived this response letter to the editor. MLTB wrote the first draft of the letter. JS, MGN and AV revised the letter critically. All authors contributed to and approved the final letter.

## Declaration of Competing Interest

The authors declare no conflicts of interest.
